# Improving the Effective Spatial Resolution in ^1^H-MRSI of the Prostate with Three-Dimensional Overdiscretized Reconstructions

**DOI:** 10.3390/life13020282

**Published:** 2023-01-19

**Authors:** Carlijn J. A. Tenbergen, Loreen Ruhm, Sjoerd Ypma, Arend Heerschap, Anke Henning, Tom W. J. Scheenen

**Affiliations:** 1 Department of Medical Imaging, Radboud University Medical Center, 6525 GA Nijmegen, The Netherlands; 2 High-Field MR Center, Max Planck Institute for Biological Cybernetics, 72076 Tübingen, Germany; 3 Advanced Imaging Research Center, University of Texas Southwestern Medical Center, Dallas, TX 75390, USA

**Keywords:** magnetic resonance spectroscopic imaging, spatial resolution, voxel bleeding, overdiscretized reconstruction, prostate imaging

## Abstract

In in vivo ^1^H-MRSI of the prostate, small matrix sizes can cause voxel bleeding extending to regions far from a voxel, dispersing a signal of interest outside that voxel and mixing extra-prostatic residual lipid signals into the prostate. To resolve this problem, we developed a three-dimensional overdiscretized reconstruction method. Without increasing the acquisition time from current 3D MRSI acquisition methods, this method is aimed to improve the localization of metabolite signals in the prostate without compromising on SNR. The proposed method consists of a 3D spatial overdiscretization of the MRSI grid, followed by noise decorrelation with small random spectral shifts and weighted spatial averaging to reach a final target spatial resolution. We successfully applied the three-dimensional overdiscretized reconstruction method to 3D prostate ^1^H-MRSI data at 3T. Both in phantom and in vivo, the method proved to be superior to conventional weighted sampling with Hamming filtering of k-space. Compared with the latter, the overdiscretized reconstructed data with smaller voxel size showed up to 10% less voxel bleed while maintaining higher SNR by a factor of 1.87 and 1.45 in phantom measurements. For in vivo measurements, within the same acquisition time and without loss of SNR compared with weighted k-space sampling and Hamming filtering, we achieved increased spatial resolution and improved localization in metabolite maps.

## 1. Introduction

Multiparametric MRI (mpMRI) of the prostate has emerged as a reliable non-invasive imaging modality for identifying clinically significant cancer, enabling selective sampling of high-risk regions with MRI-targeted biopsies, aiding in therapeutic planning and active surveillance [1,2]. A clinical mpMRI examination consists of the acquisition of T_2_-weighted (T2w) images, DWI, and a gadolinium-based DCE series, deriving both anatomical and functional information from the prostate. It has been suggested that combining mpMRI with blood-sampled PSA-derived biomarkers could relieve uncertainty in the initial diagnosis [3]. Radiomics in prostate cancer, focused on quantitative data from, e.g., MRI, could improve assessments of prostate cancer risk categories at initial diagnosis, allowing a personalized treatment approach [4,5].

Another quantitative MRI modality, proton magnetic resonance spectroscopic imaging (^1^H-MRSI), has also been shown as useful because healthy and cancerous prostate tissue regions present different choline and citrate signal intensities [6,7,8]. Ratios including these metabolites in combination with anatomical T2w MR imaging can identify and localize prostate cancer [9]. Previous work in a multi-center setting has also shown a correlation between the metabolite ratios and prostate cancer aggressiveness classes [10], indicating a role for ^1^H-MRSI in characterizing prostate cancer.

Clinical use of prostate spin–echo ^1^H-MRSI methods, however, is hindered by its limited robustness. Challenges in acquisition and post-processing techniques require special expertise [11] and the relatively long acquisition time of 5 to 10 min might outweigh the additional value to routine multiparametric MRI [12]. Next to these long acquisition times, the large voxel sizes of approximately 0.9 cm^3^ to gain a sufficient metabolite signal in MRSI is regarded as a drawback. This low spatial resolution causes voxel bleeding to extend over larger parts of the prostate. Voxel bleeding is apparent from the side lobes of the spatial response function (SRF), describing the origin of the signal for a specific voxel of the MRSI matrix, which extends all over the field of view (FOV) of the MRSI grid [13]. This spread of signal hinders precise localization of the signals of interest and causes the interference of other metabolites in the region of interest, such as peri-prostatic residual lipid signals extending into prostate tissue.

The side lobes of the SRF can be suppressed by applying a Hamming filter to the k-space data before spatial Fourier transformation (FT), decreasing far-reaching voxel bleeding [14]. In combination with the weighted sampling of k-space, this is currently often used as an ^1^H-MRSI acquisition strategy [15]. Applying this filter comes at the cost of an increase in the effective voxel size, seen as a widening of the SRF main lobe, leading to an even lower spatial resolution of the resulting metabolite maps [14].

As an alternative to weighted sampling, an overdiscretized SRF target-based reconstruction method was presented by Kirchner et al. [16]. Developed as an improved SENSE reconstruction method [17] for single-slice two-dimensional (2D) ^1^H-MRSI brain data, the method was shown to be effective in suppressing near and far-reaching voxel bleed and correcting for intra-voxel coil sensitivity variations. In later work, a noise decorrelation was added to the method to correct for intra-voxel frequency shifts, improving spectral line shapes and increasing the signal-to-noise ratio (SNR) in the presence of the main magnetic (B0) field inhomogeneities on top of the voxel bleeding effects [18]. These methods were applied to a single brain slice and limited to a spatial 2D field of view. The potential to extend the application to other regions within the human body studied with 3D-MRSI has not yet been explored.

In the prostate, a small organ in the center of the lower abdomen, the B0 field is mostly quite homogeneous, with occasional B0 field distortions at the posterior edge of the prostate resulting from the presence of rectal gas [19]. Without large intra-voxel B0 inhomogeneities, the method of noise decorrelation with intra-voxel frequency shifts using B0 field maps is not expected to improve the spectra too much. We propose the use of random frequency shifts for the noise decorrelation of the spectra before the last step of applying a target function to reconstruct the final voxel sizes.

In this work we present the development of a three-dimensional overdiscretized and target-based MRSI reconstruction method (3D ODR method) to improve the spatial resolution of the 3D volume-selective MRSI of the prostate. After the initial overdiscretization of the MRSI grid, we used random frequency shifts for noise decorrelation before the last step of applying a target function. We compared this method with the conventional 3D MRSI with weighted k-space sampling and Hamming filtering of k-space at 3 Tesla. Without increasing the acquisition time from current 3D MRSI acquisition methods, the aim is to improve the localization of metabolite signals in the prostate without compromising on SNR.

## 2. Materials and Methods

### 2.1. Theory

The overdiscretized reconstruction method consists of three steps: overdiscretization of the MRSI grid, noise decorrelation between the subvoxels, and application of a target function to return to the final spatial resolution [16,18,20]. For the current 3D volumetric application, multiple single-slice operations in the existing 2D approach were replaced by volumetric reconstructions, while parallel imaging was omitted, coil elements were combined by the scanning system, a spin–echo sequence is used, and the method of noise decorrelation is revised.

The reconstruction method starts with overdiscretizing the MRSI grid in k-space by zero-padding the original grid (of N voxels). This creates an intermediate grid with, after 3D FT, an increased interpolated spatial resolution. The size of the intermediate grid is determined by the choice of the overdiscretization factor (ζ). The same factor was applied to all three dimensions, resulting in a ζ^3^-fold overdiscretized spatial grid. In target-driven overdiscretized reconstruction [20], a minimization is performed of the cost function (Δ) for each voxel:

(1) $$\Delta {=\|(\mathrm{FE}}_{\mathrm{OD}}{\;-\;T)\|}_{2}^{2}$$

where F, $E_{\mathrm{OD}}$, and T are the reconstruction operator, the overdiscretized encoding matrix, and the target matrix, respectively. The analytical solution of the optimization problem is given by:

(2) $${F=\mathrm{TF}}_{\mathrm{OD}}$$

with

(3) $$F_{\mathrm{OD}}{=(E}_{\mathrm{OD}}^{H}{\;E}_{\mathrm{OD}}{)}^{†}{\;E}_{\mathrm{OD}}^{H}\;$$

in which $F_{\mathrm{OD}}$ reconstructs to the intermediate spatial grid of ζ^3^N subvoxels. After applying $F_{\mathrm{OD}}$ on the zero-padded k-space data s_OD_,

(4) $$\rho_{\mathrm{OD}}{=F}_{\mathrm{OD}}{\;s}_{\mathrm{OD}}$$

the noise of the spectra’s neighboring subvoxels in the intermediate grid ρ_OD_ are strongly correlated. This correlation can be decreased, as the second step in the reconstruction, by a per-voxel frequency shift of the spectra (Δf(**r**)) of the subvoxels at locations **r**, resulting in a corrected intermediate grid:

(5) $$\rho_{\mathrm{OD}}^{\mathrm{corr}}{=\rho }_{\mathrm{OD}}{(r,\;t)e\;}^{-i2\pi \Delta f(r)t}$$

The frequency shifts per voxel for the noise decorrelation were constructed with a map with random spatially distributed shift factors applied on the intermediate grid of the subvoxels. With the random spatial distribution of spectral shift factors, it is ensured that the noise between subvoxels is decorrelated, decreasing noise after weighted summation with a target function in the next step. With the number of spectral points to shift chosen within a small range (ranging from minus 2 to plus 2 spectral points), the widening of metabolite peaks is constrained.

As the third and final step, a 3D target function (T) is applied to the spatial intermediate grid after noise decorrelation to act similar to a spatial averaging operator:

(6) $${\rho =T\rho }_{\mathrm{OD}}^{\mathrm{corr}}\;$$

This target function sums up spectra from the individual subvoxels in a weighted manner to return to the original grid resolution. This weighted spatial average of subvoxels, achieved by an SRF target with a 3D Gaussian shape that suppresses long-range signal bleeds, leads to the final effective voxel size of the reconstructed MRSI data, which is close to the original nominal voxel size.

**Figure 1:**
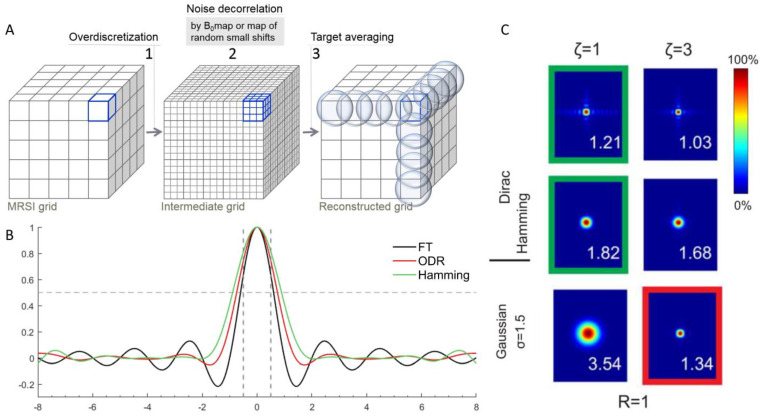
(**A**) Schematic overview of the 3D overdiscretized reconstruction method. The original MRSI grid is first transformed into a higher-resolution intermediate grid by zero-padding of k-space with factor 3. Each subvoxel spectrum is then frequency-shifted according to the corresponding B0 map or random shifts. In the third step, the target resolution and shape of overlapping spheres is achieved by a weighted average of the subvoxels. (**B**) SRF in 1D after FT of fully sampled k-space and the SRF shapes after ODR reconstruction (red) and Hamming filtering (green), visualizing the main peak and side lobes for each reconstruction. Dashed lines indicate nominal resolution width and height at half maximum. (**C**) The FWHM of the main peak of 2D SRF of a central voxel is indicated by the numbers (adjusted and reproduced with permission [16]). The factors for overdiscretization (ζ = 3) and gauss target width (σ = 1.5) of the SRF in the red box were extrapolated to 3D in this work and compared with Hamming filtering in the bottom green box.

A schematic representation of the 3D ODR method is shown in Figure 1. The reconstruction method acts on two aspects: the overdiscretization and target function improve the SRF shape and corresponding signal localization while the noise decorrelation results in improved SNR. From previous work, the combination of an overdiscretization factor of 3 and target function width (σ) of 1.5 subvoxels was adopted, as this was shown to be optimal for SRF shape improvement [16]. The SRF shape after ODR with these parameters has very low residual side lobes (Figure 1).

With the chosen overdiscretization factor 3 and subsequent averaging with the target function of width 1.5, the resulting SRF has a width at a half height of 1.34 vs. 1.82 for the Hamming-filtered data (width relative to the nominal voxel width of the measurement) [16]. These numbers can be extended from a 2D voxel diameter representation to a 3D voxel volume representation, using half this width in the formula for calculating the volume of a sphere (V = 4/3 × πr^3^). An ODR dataset would represent a voxel volume of 1.26 times a nominal cubic voxel volume, as opposed to a voxel volume of 3.16 times the nominal volume for a conventional weighted dataset with Hamming filtering.

To illustrate the benefits of the improved SRF shape by our 3D ODR method we performed phantom measurements to analyze the effect of the reconstruction method on signal localization and measurements on a healthy volunteer to assess the feasibility in vivo for the application to ^1^H-MRSI of the prostate.

### 2.2. Acquisition

All measurements were performed on a Siemens 3T MR system (MAGNETOM Prisma-fit, Siemens Healthineers AG, Erlangen, Germany) using an 18-channel body phased-array coil and a 32-channel spine phased-array coil for signal reception (Siemens Healthineers AG, Erlangen, Germany). T2w images, used as reference and anatomical background, were acquired in three directions by a TSE sequence (TE = 101 ms, 2 averages; Tra: TR = 5660 ms, 0.3 × 0.3 × 3.0 mm; Sag: TR = 5590 ms, 0.6 × 0.6 × 3.0 mm; Cor: TR = 5000 ms, 0.6 × 0.6 × 3.0 mm). We acquired 3D ^1^H-MRSI with a GOIA semi-LASER ^1^H-MRSI sequence [21] with scan parameters: TR = 750 ms, TE = 88 ms, spectral bandwidth = 2.4 kHz, and 1024 time samples. As the signal from citrate, the most abundant metabolite in healthy prostate, originates from a strongly coupled spin system, its spectral shape depends on sequence timing. For the semi-LASER sequence, the echo time of 88 ms and corresponding pulse timing results in an optimal signal shape of citrate in the prostate [21].

**Figure 2:**
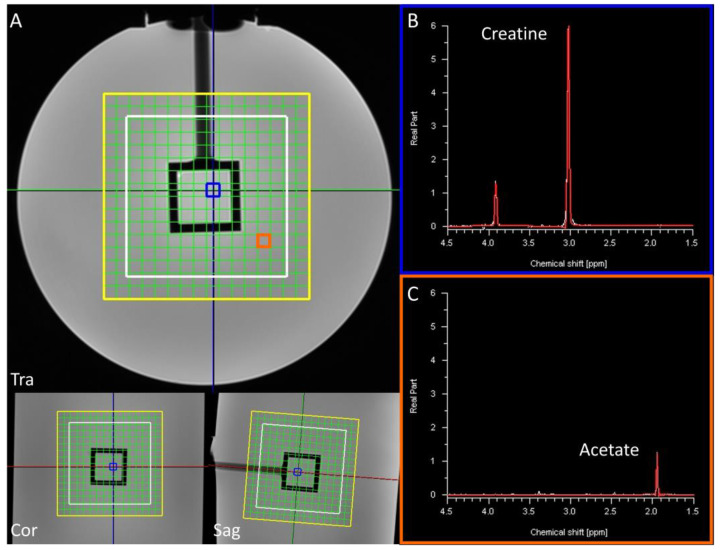
The phantom used in this study contains a creatine solution in the inner cube (spectrum from blue voxel, panel (**B**)) and acetate in the surrounding cylinder (spectrum from orange voxel, panel (**C**)). The matrix grid is indicated in yellow; the VOI is outlined in white (**A**). The colored lines in panel (**A**) show each plane in the other dimensions. The main peak of creatine (at 3.03 ppm) was set to represent the signal inside of the central cube and the integral of its fit was used to make creatine maps.

The phantom used in this study is a cylindrical container filled with a sodium acetate solution with a cubic inner compartment with a creatine solution (Figure 2). This box-shaped inner compartment has distinct straight edges and can therefore be used for localization analyses. The singlet of creatine at 3.04 ppm in the inner cube is the signal of interest and the ^1^H-MRSI is acquired with a matrix size of 11 × 11 × 11 voxels and FOV of 77 × 77 × 77 mm^3^. We used two acquisition approaches: one intended for ODR, in which k-space is fully sampled in an acquisition time of 16:40 min with 1 average; the other, in which k-space is sampled in a Hamming-weighted manner [14] with 8 k-space center averages to approximate a similar acquisition time (15:21 min). With these settings, both acquisitions yielded a similar acquisition time and matrix.

The acquisition strategy above does not result in equal voxel sizes for both datasets, as the width of the main lobe in the SRF is different for the weighted sampling and Hamming filtering versus the full k-space sampling with ODR. A second set of phantom measurements was aimed at acquiring similar final voxel sizes for both acquisition strategies within the same acquisition time. The mentioned SRF sizes, 1.34 vs. 1.82 times the radius of the spherical voxel for full k-space sampling with ODR and for weighted k-space sampling and Hamming filtering, respectively, were used to set up matrices resulting in equal voxel sizes. The first ^1^H-MRSI acquisition with a full sampling of k-space was performed with a matrix of 11 × 11 × 11 voxels, FOV of 88 × 88 × 88 mm^3^, and 2 averages in 28:52 min (voxel radius = 0.5 × 8 × 1.34 = 5.36 mm). The subsequent acquisition with weighted sampling of k-space was performed with 6 k-space center averages on a matrix of 15 × 15 × 15 voxels, with an FOV of 90 × 90 × 90 mm^3^ and acquisition time of 30:23 min (voxel radius = 0.5 × 6 × 1.82 = 5.46 mm).

Next to the two sets of phantom measurements, datasets from a healthy 45-year-old male volunteer, obtained in accordance with local ethics regulations, were included. No preparation to suppress peristaltic motion was performed. First acquisition with full k-space sampling (1 average) was acquired in 11:10 min with a matrix size of 11 × 9 × 9 voxels, FOV 88 × 72 × 72 mm^3^, MEGA water, and fat suppression. A second acquisition with weighted k-space sampling was acquired with 9 averages and an acquired FOV size of 77 × 63 × 63 mm^3^ (matrix size 11 × 9 × 9) in an acquisition time of 11:04 min. With these measurements, a similar acquisition time and matrix were achieved for both acquisitions.

### 2.3. Reconstruction

The weighted datasets were reconstructed by Hamming filtering of k-space, followed by zero-filling to a matrix of 16 × 16 × 16 voxels and spatial FT. This dataset functioned as a standard for comparison with the ODR pathway. The fully sampled datasets were zerofilled to 16 × 16 × 16 matrices and were processed by the 3D overdiscretized reconstruction method in Matlab (The Mathworks, Natick, MA). For the first step, an overdiscretization factor of 3 increased the MRSI grid of 16 × 16 × 16 voxels into an intermediate MRSI grid of 48 × 48 × 48 subvoxels. Noise decorrelation was subsequently performed per voxel on the high-resolution intermediate grid. Random spatial distributions of shift factors were simulated with the same resolution, e.g., 48 × 48 × 48, and frequency shifts were imposed on corresponding subvoxels. Shift factors were chosen in the range from −2 to 2 spectral points to ensure noise decorrelation while restraining spectral peak broadening. The noise-decorrelated intermediate spectroscopic image was transformed to the target resolution of 16 × 16 × 16 voxels by the SRF target function with σ = 1.5 subvoxels, as shown successfully in combination with the overdiscretization factor 3 in previous 2D work [16].

### 2.4. Signal Analysis

The data processed with 3D ODR in Matlab and the originally acquired and postprocessed data (3D Hamming filter of zero-filled k-space) were imported in syngo.via (Siemens Healthineers AG, Erlangen, Germany) and all fitted with the same prior knowledge. For the phantom datasets, the signals of creatine (3.03 ± 0.05 ppm and 3.91 ± 0.05 ppm), lactate (1.34 ± 0.05 ppm), and acetate (1.9 ± 0.1 ppm) were fitted with a Gaussian function. The main peak of creatine (at 3.03 ppm) was chosen to represent the signal inside of the central inner cube and the integral of its fit was used to make metabolite maps. Creatine maps were normalized to the average integral value of the 8 voxels in the center of the cube. The creatine maps for the ODR data and Hamming-filtered data were compared both side to side as well as after subtracting one from the other in a difference map for a quantitative analysis of differences in voxel bleed. SNR was defined as the peak integral of creatine per voxel divided by the noise, stated as the standard deviation of the signal in an area of the spectrum not containing metabolites. To compare SNR values between the datasets with different voxel sizes, only voxels completely within the inner cube were chosen. These center 8 voxels overlapped with each other; the SNR values were therefore not independent. As the SNR is determined by both the SRF shape and the frequency shift, the separate contributions will be further analyzed by rendering an additional dataset for each experiment of full k-space sampling by applying ODR without the per-voxel frequency shift.

For the in vivo datasets, signals from citrate and residual lipids were fitted with appropriate line shapes at 2.6 ± 0.1 ppm and 2.3 ± 0.2 ppm, respectively. For the combination of total choline (3.21 ppm), polyamines (spermine at 3.12 ppm), and creatine (3.03 ppm) signals, the individual metabolite peaks were fitted as separate peaks. Metabolite maps of citrate and lipids were created and used for a qualitative analysis of signal localization. SNR values were extracted from the integral of the citrate peak over the noise value, where noise was again defined as the standard deviation of the signal in an area of the spectrum not containing metabolites. To compare spectral quality between ODR with and without frequency shift and Hamming-filtered data, SNR values from all datasets were compared in a voxel-wise manner, with a correction to account for the difference in relative voxel sizes. A selection of voxels from the same spatial location in all datasets, which were completely within the prostate with minimal overlap with each other, were chosen for SNR comparison. Localization of metabolite signals in both datasets was qualitatively analyzed by comparing citrate and lipids maps.

## 3. Results

To analyze the effects of the developed 3D ODR method on 3D ^1^H-MRSI data, we assessed signal localization and the SNR of the methodology by a comparison with the traditional reconstruction with Hamming filtering.

### 3.1. Signal Localization

**Figure 3:**
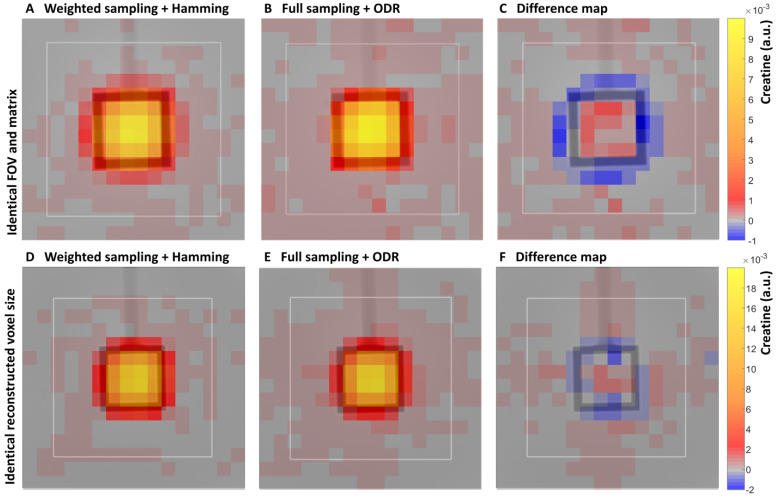
A phantom comparison of traditional sampling and ODR reconstruction. (**A**) Representative creatine intensity maps (in a.u.) of a center partition of the 3D datasets for weighted k-space sampling and Hamming filtering (matrix size: 11 × 11 × 11, FOV: 77 × 77 × 77 mm^3^). (**B**) Full k-space sampling and ODR reconstruction (matrix size: 11 × 11 × 11, FOV: 77 × 77 × 77 mm^3^). (**C**) Difference map (**A**,**B**) with same scaling shows localization of signal differences between datasets. The bottom row displays creatine maps from the second phantom acquisition aimed at equal voxel sizes: (**D**) weighted k-space sampling and Hamming filtering, (**E**) full k-space sampling and ODR reconstruction. (**F**) Difference (**D**,**E**) between the datasets is displayed with same scaling. Note that the creatine maps were separately normalized to the intensity of the center 8 voxels within the cube.

Signal localization for the two reconstruction methods was assessed by metabolite maps. The distribution of the creatine signal over the phantom was displayed in creatine maps with one partition of the 3D dataset in one orientation, representative of partitions through the center of the phantom in the other two directions (Figure 3). The creatine maps showed high signal intensity in the center of the inner cube of the phantom and decreasing intensity in voxels towards and over the edge of the cube for the weighted Hamming-filtered dataset. For the ODR dataset, this decrease in signal along the edges of the inner cube was more distinct, visualizing less voxel bleeding. This was due to the smaller main peak in the SRF, which resulted in a smaller effective voxel size for the ODR dataset compared with the weighted Hamming-filtered dataset. The difference map showed that this difference in voxel bleeding accounted for up to 10% of the normalized signal intensity.

In the subsequent phantom dataset, where scan parameters were set up to acquire equal voxel sizes, the weighted k-space sampled and Hamming-filtered dataset was subtracted from the fully sampled k-space with the ODR dataset. In this difference map, the intensity in the center voxels was in the same range as background noise, so there was indeed no distinct difference in localization when the two acquisitions were matched in effective voxel size.

A direct comparison between the two reconstruction approaches was made in all three orientations of the first phantom measurement (difference maps in Supplementary, Figure S1). The ODR dataset contained higher creatine signal intensities in the center cube of the phantom in comparison with the Hamming dataset. Negative values outside of the cube showed lower creatine levels in these voxels in the ODR dataset, which was indicative of improved signal localization ranging from 5 to 10% in all three directions in the ODR dataset, improving 3D SRF shape and signal localization.

**Figure 4:**
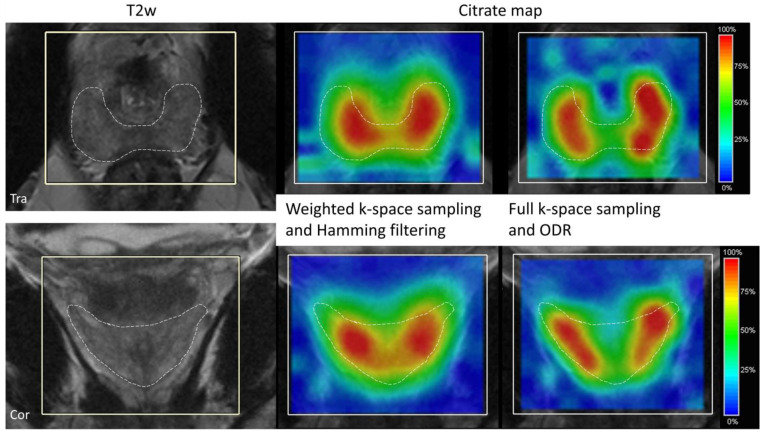
Citrate maps in transversal view (**top**) and coronal view (**bottom**) of a volunteer for the weighted k-space sampling and Hamming filtering (matrix size: 11 × 9 × 9, FOV: 77 × 63 × 63 mm^3^, 9 averages in 11:04 min acquisition time) (**middle**) and full k-space sampling and ODR (**right**) acquisition (matrix size: 11 × 9 × 9, FOV: 88 × 72 × 72 mm^3^, 11:10 min acquisition). Outlined is the peripheral zone of the prostate, where most of the citrate signal can be expected.

**Figure 5:**
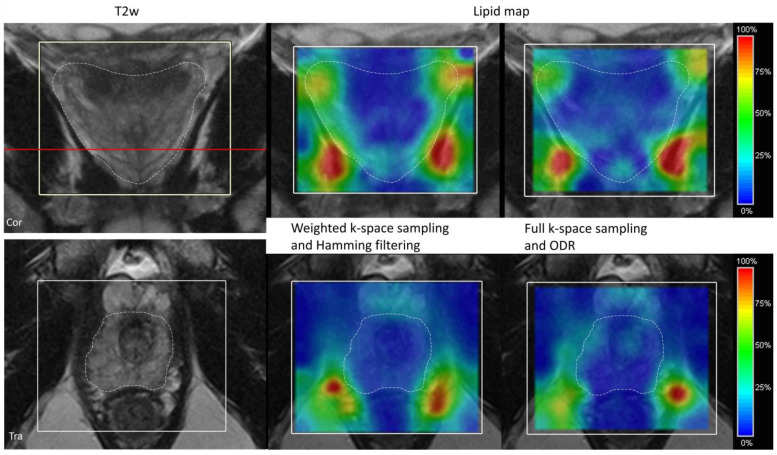
Lipid maps in coronal (**top**) and transversal (**bottom**) view of the volunteer for the weighted k-space sampling and Hamming filtering (matrix size: 11 × 9 × 9, FOV: 77 × 63 × 63 mm^3^, 9 averages in 11:04 min acquisition time) (**middle**) and full k-space sampling and ODR (**right**) (matrix size: 11 × 9 × 9, FOV: 88 × 72 × 72 mm^3^, 11:10 min acquisition). The dashed line indicates the edges of the prostate. The red line in the coronal view indicates the position of the transversal plane.

Improved signal localization for the 3D ODR was also seen in the results from the volunteer measurement with representative partitions of the 3D citrate map in two orientations for the Hamming-filtered weighted dataset and fully sampled ODR dataset (Figure 4). The citrate signal in the ODR dataset was more confined to the healthy peripheral zone, with steeper edges and less bleed of signal along the edges outside of the prostate. When visualizing the spatial distribution of lipids around the prostate (Figure 5), a representative coronal slice of the lipid map shows less lipid contamination in the ODR dataset as the amount of signal originating from lipids in the surrounding tissues is decreased in the prostate tissue, as seen in the anatomical reference T2w image.

### 3.2. SNR

To assess whether the 3D ODR method compromises on SNR, corresponding voxels from both reconstruction methods were compared. The first phantom measurements resulted in an average SNR value in the central eight voxels of 8.30 (a.u.) for the ODR dataset, and 11.1 for the Hamming-filtered dataset (Table 1). The ODR dataset in the central eight voxels thus contains 0.75 times the SNR of the Hamming-filtered data, without corrections for corresponding voxel sizes. Correcting for the SRF-derived normalized calculated spherical voxel volumes (ODR: 1.26 vs. Hamming: 3.16 vs. hypothetical 1 × 1 × 1 cube), the SNR for the ODR dataset in comparison to the Hamming-filtered dataset was increased on average with a factor of 1.87.

For the second phantom measurements, the SNR for the ODR dataset in comparison with the Hamming-filtered dataset is increased with a factor of 1.45 on average. While the voxel sizes were matched between the datasets, the center eight voxels indicated an SNR gain for the ODR.

**Table 1:** SNR results for the three different experiments. The last column shows the ratio of SNR value for the ODR dataset over the weighted sampling and Hamming-filtered dataset, corrected for relative voxel sizes.

		Effective Resolution	Voxel Radius	Mean SNR	Mean SNR Size Corrected	Ratio to Weighted Sampling + Hamming
phantom 1	Full k-space sampling	8 × 0.5 × 1.34	5.36	24.81	3.85	N/A
	Weighted sampling + Hamming filter	8 × 0.5 × 1.82	7.28	111.1	6.88	N/A
	ODR	8 × 0.5 × 1.34	5.36	83.02	12.87	1.87
	ODR w/o frequency shift	8 × 0.5 × 1.34	5.36	36.90	5.72	0.83
phantom 2	Full k-space sampling	8 × 0.5 × 1.34	5.36	35.57	N/A	N/A
	Weighted sampling + Hamming filter	6 × 0.5 × 1.82	5.46	84.56	N/A	N/A
	ODR	8 × 0.5 × 1.34	5.36	122.5	N/A	1.45
	ODR w/o frequency shift	8 × 0.5 × 1.34	5.36	51.44	N/A	0.61
volunteer	Full k-space sampling	8 × 0.5 × 1.34	5.36	5.26	0.81	N/A
	Weighted sampling + Hamming filter	7 × 0.5 × 1.82	6.37	20.6	1.90	N/A
	ODR	8 × 0.5 × 1.34	5.36	14.1	2.19	1.15
	ODR w/o frequency shift	8 × 0.5 × 1.34	5.36	6.79	1.05	0.55

**Figure 6:**
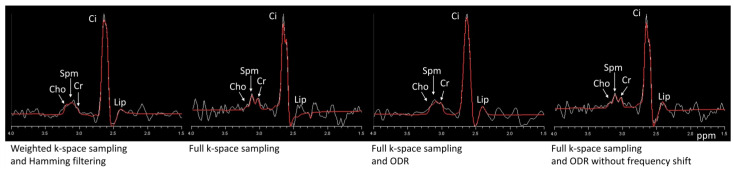
Spectra of a voxel within the peripheral zone of the prostate for the weighted k-space and Hamming filter dataset (**left**), next to a subsequently fully sampled dataset, full k-space sampling with ODR, and full k-space sampling with ODR without the frequency shift (**right**).

The representative spectra of the volunteer measurement are shown in Figure 6. The dataset with weighted sampling and Hamming filtering showed clear and smooth peaks and low noise. The original fully sampled dataset had a high noise level, and by applying the ODR without the frequency shift, it decreases this noise due to the slight broadening of the main lobe of the SRF. In the case of ODR, including the frequency shift, the spectrum showed less high-frequency noise. The line broadening that is visible as a loss of some spectral resolution in the resonance groups of choline, spermine (polyamines), and creatine is comparable to the dataset of the weighted sampling and Hamming filtering. These three resonances often overlap in prostate MRSI, and assessment of individual linewidths in these groups is very challenging. In citrate, a strongly coupled spin system, line broadening only caused a loss of separation of the two center peaks of the spectral shape, hardly affecting the total appearance of the total citrate signal.

Comparing the ODR dataset with the Hamming-filtered dataset in eight independent voxels from the peripheral zone, the mean SNR values were 14.1 and 20.6 (a.u.), respectively. Thus, the ODR reached 0.68 times the SNR of the Hamming-filtered data. Correcting for the difference in voxel size, this translated to an increase in SNR with a factor of 1.15 for the ODR dataset compared with the Hamming-filtered dataset.

From Table 1 it can be appreciated that for all datasets, applying ODR without a frequency shift does improve SNR compared with the original full-sampling dataset because of SRF main-lobe broadening and side-lobe suppression. The ODR, including the frequency shift, however, combines SRF improvement and noise decorrelation, showing the mentioned gain in SNR compared with the Hamming-filtered datasets.

## 4. Discussion

In this work, we developed a 3D overdiscretized reconstruction method for semi-LASER 3D ^1^H-MRSI and applied it to phantom and in vivo prostate measurements. With the 3D ODR method, it is possible to suppress far-reaching voxel bleed, with less increase in the effective voxel size as with weighted k-space sampling and Hamming filtering. In addition to the superior signal localization, a moderate gain in SNR was achieved.

Spatial resolution is an important aspect in MRSI, as a relatively large voxel size is needed to acquire signals from metabolites instead of water. This gives rise to partial volume effects, with one voxel representing a signal from different tissues, as is visible in the SRF, which hinders detecting or characterizing small lesions. With Hamming filtering, spatial resolution is traded for the suppression of far-reaching voxel bleed, with a widened main lobe of the SRF and decreased side lobes. The 3D ODR method increases the true spatial resolution of metabolite maps in comparison with Hamming filtering, matching underlying anatomical structures as found on clinical prostate MR imaging, and may therefore improve the metabolic characterization of small prostate cancer lesions.

For the second step of the ODR method, noise decorrelation of the overdiscretized 3D MRSI grid, two strategies can be used. The initial overdiscretization into an intermediate grid is created in real space by the zero-filling of the acquired k-space. Overlap of the respective SRFs is the major source of noise correlation in neighboring subvoxels of the intermediate grid. Imposing a small random frequency shift on the subvoxels before the third step of applying a target function to return to the final spatial resolution leads to noise decorrelation. If noise decorrelation is based on measured B0 maps, the frequency alignment of subvoxels can improve linewidths. However, with noise decorrelation based on random spectral shifts, instead of using a B0 map, spectral line widths can increase [18]. In phantom measurements with well-resolved and very narrow spectral lines, this spectral line broadening is visible. Choosing only few random spectral points to shift relative to the linewidth of the metabolites of interest prevents strong line broadening. Linewidths were larger in measurements of the prostate than in the phantom, not only because of tissue susceptibility differences but possibly also due to small body movements [22]. Noise decorrelation in the intermediate grid was based on random spectral point shifts between −2 and +2 points. The resulting spectra from the volunteer measurements showed some peak broadening. However, its impact on metabolite analysis is limited with the usual processing of quantifying the total spectral integral from 3.21 to 3.04 ppm with the sum of three spectral shapes of choline, spermine, and creatine, and with only a loss of the splitting of the two center peaks of the strongly coupled spectral shape of citrate.

Application of the ODR method, both to the phantom and volunteer measurements, showed an improved localization of signals of interest and decreased lipid contamination within the prostate of the volunteer. Metabolite localization and SNR could be directly compared in the phantom between weighted k-space sampling with Hamming filtering and full k-space sampling with ODR, as the metabolite concentration and localization within the inner cube of the phantom was homogeneous and demarcated. The resulting difference maps in all three dimensions visualized the improved signal localization as a result of the improved 3D SRF shape when measuring with the same acquisition matrix in the same acquisition time. For the volunteer measurements, overlaying both datasets on anatomical T2w images provided the location of the peripheral zone or whole prostate gland as boundaries for estimated signal localization. However, comparing the signal intensities of voxels with different sizes remains challenging, as tissue metabolite signal intensity is not necessarily homogeneous, with varying metabolite concentrations across the whole prostate. Some studies have shown the use of anatomical boundaries to constrain metabolite signals within these boundaries, with the risk of biases in quantifying differences in metabolite signals within this anatomical region [23]. Apart from voxel definition by the two ways of MRSI data processing, accurate localization of metabolite signal within the whole volume of interest is also due to the use of a semi-LASER pulse sequence in the ^1^H-MRSI acquisition of the phantom and volunteer. This volume selection already provides more accurate localization over a PRESS sequence for the whole VOI [21].

The effect of ODR on the observed SNR in the two separate phantom measurements needs clarification. An increase in SNR of 1.87 was achieved for the fully sampled and ODR dataset compared with the weighted k-space sampling and Hamming filtering data in the first measurement. This measurement involved different voxel sizes and a factor to correct for this choice. In the second phantom measurement we aimed at achieving a similar voxel volume for both the ODR dataset and the weighted Hamming-filtered dataset. Matching final voxel sizes was successful, as could be seen from the localization difference image between the two acquisitions (Figure 3F). The ODR method resulted in a SNR increase of 45%. In earlier work, Nassirpour et al. [20] found an average 23% SNR gain in ODR data with SENSE compared with reconstructing with SENSE alone (with ζ = 4 at a field strength of 9.4T). Kirchner et al. [18] found an increase in SNR between the ODR data and the Hamming-filtered data with a factor of 3.7 (with ζ = 5 at a field strength of 7T). There are several differences between those previous studies and the current one, and next to field strength and B0 homogeneity in the FOV, the spatial resolution of the MRSI datasets, the exact definition of SNR (peak or integral), and the upgrading to 3D can influence the SNR gain.

For the SNR comparison in the first phantom measurements, we calculated 3D voxel sizes extrapolated from simulated 2D SRF values, assuming spherical voxel sizes with a diameter of FWHM of the SRF. Moving from a 1D sinc-like, to a 2D circular shape, to a 3D spherical SRF facilitates calculations for effective voxel volume in 3D from the simulated numbers for 1D and 2D. The effective final voxel volumes were subsequently used as the correction factor for SNR comparison. SRF line shape differences can cause differences in assumed voxel volumes between the two phantom measurements, resulting in different values for the correction factor for both signal and noise in the SNR of different voxel volumes.

In the datasets from the volunteer examination, it is more difficult to assess SNR gain. As the two datasets are of different spatial resolutions, one cannot choose the same position and voxel size in the two acquisitions; therefore, local tissue concentration differences can obscure a fair SNR comparison (even with correction for voxel size). SNR averaged over voxels completely within the peripheral zone was the best option here to compare both datasets, with full k-space sampling with ODR exceeding the SNR of the metabolites of interest over weighted k-space sampling with Hamming filtering.

Subsequent testing of the ODR method in patients with prostate cancer can establish the effect of improved signal localization and SNR for tumor characterization. With applying ODR on a fully sampled k-space dataset, the spatial resolution acquired in our in vivo examination required an acquisition time of 11 min. With expected differences in prostate sizes and larger prostate VOIs in older subjects, the acquisition time for MRSI might become too long. A solution would be to combine complete k-space sampling and ODR with accelerated read-outs, e.g., EPI or spiral read-out methods [24].

With an accelerated acquisition and automated post-processing workflow, resulting high-quality spectra could be of additional value in the clinical management of prostate cancer. Especially in repeated examinations per patient, as in active surveillance, we believe acquiring quantitative MRSI data enables direct comparison between scans and maximizes non-invasively acquired information of the prostate.

## 5. Conclusions

In this study, we successfully developed and implemented a three-dimensional overdiscretized reconstruction method for semi-LASER 3D MRSI data of the prostate. Both in phantom and in vivo, the method proved to be superior in spatial resolution to conventional weighted sampling with Hamming filtering of k-space. Compared with the latter, data after overdiscretized reconstruction have a smaller voxel size while maintaining higher SNR. With an improved localization of metabolites in a phantom measurement and in vivo, the 3D ODR method is a promising tool to increase the quality of spectra and the spatial resolution of metabolite maps and hence their match to underlying anatomical structures.

## Data Availability

Datasets used and/or analyzed during the current study are available from the corresponding author upon reasonable request.

## Supplementary Materials

The following supporting information can be downloaded at: https://www.mdpi.com/article/10.3390/life13020282/s1, Figure S1: Creatine difference maps in three dimensions.
