# Supplementary material for: Improving the Effective Spatial Resolution in 1H-MRSI of the Prostate with Three-Dimensional Overdiscretized Reconstructions
Source: Life (Basel). 2023 Jan 19;13(2):282. doi: 10.3390/life13020282 (PMC9967259; doi:10.3390/life13020282)

Supplementary Figure S1: Creatine difference maps from phantom acquisitions displayed in three dimensions. After subtracting the weighted and Hamming-filtered k-space dataset from the fully k-space sampled and ODR dataset, the difference between these creatine maps is expressed as a percentage. Voxels in red indicate higher signal in the ODR dataset, mainly inside of phantom inner cube (dashed line), while blue indicates higher signal intensity in the weighted k-space and Hamming filter dataset, along the edges outside of phantom. Signal intensities are normalized per dataset to total signal over the FOV.

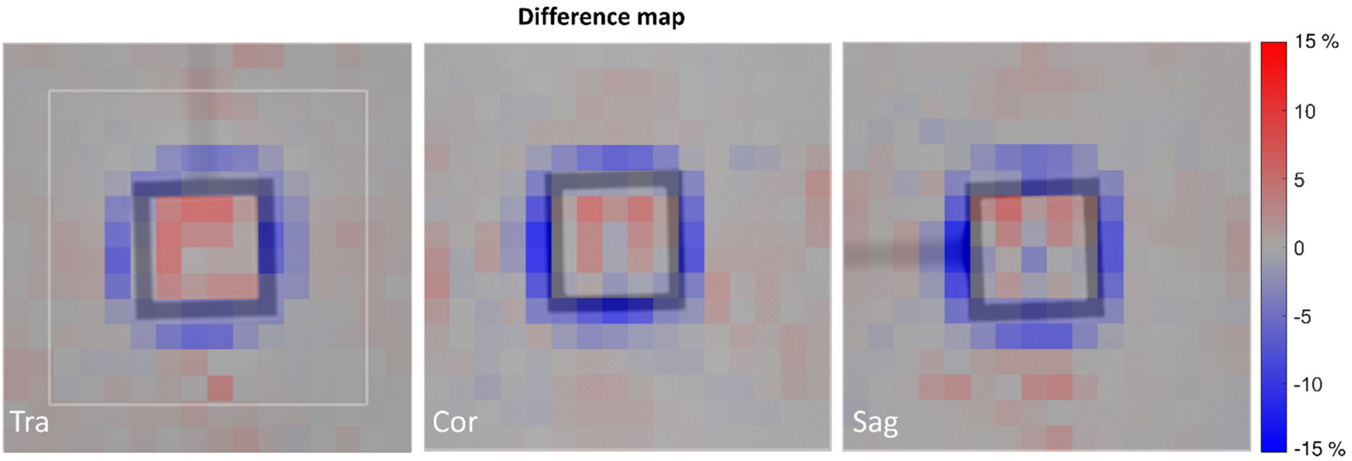

Supplement: Supplementary file 1 [file life-13-00282-s001.zip › life-2125523-supplementary.pdf]
